# Febrile neutropenia caused by the rare organism *Phytobacter*: first Case Report from India

**DOI:** 10.3389/fmed.2026.1735688

**Published:** 2026-03-03

**Authors:** Anusha Mruthyunjaya Swamy, Prasenjit Das, Indrani Sarkar, Deepak Sundriyal, Amber Prasad, Saugata Hazra, Uttam Kumar Nath

**Affiliations:** 1 Department of Medical Oncology Haematology, All India Institute of Medical Sciences, Rishikesh, Uttarakhand, India; 2 Department of Microbiology, All India Institute of Medical Sciences, Rishikesh, Uttarakhand, India; 3 Department of Biosciences and Bioengineering, Indian Institute of Technology, Roorkee, Uttarakhand, India; 4 Centre for Nanotechnology, Indian Institute of Technology, Roorkee, Uttarakhand, India

**Keywords:** febrile neutropenia, immune-compromised, nosocomial, *Phytobacter*, sepsis

## Abstract

**Background:**

*Phytobacter diazotrophicus* is an emerging opportunistic, Gram-negative bacterium, originally recognized as a nitrogen-fixing, plant-associated organism and increasingly implicated in nosocomial infections. We report the first documented case of bloodstream infection due to *P*. *diazotrophicus* in an elderly female breast cancer patient with chemotherapy-induced febrile neutropenia.

**Case presentation:**

A 62-year-old woman with HER2-positive, cT4bN2M0 breast cancer receiving neoadjuvant trastuzumab, carboplatin, and docetaxel presented with fever, headache, profound fatigue, pallor, and retinal hemorrhages. Laboratory evaluation revealed severe pancytopenia, with a platelet count of 5 × 10⁹/L, an absolute neutrophil count of 0.294 × 10⁹/L, a total leukocyte count of 1.05 × 10⁹/L, and a hemoglobin level of 3.7 g/dL. Blood cultures grew non-lactose-fermented Gram-negative bacilli, initially identified as *Pantoea* species by the VITEK-2 system; however, 16S rDNA sequencing confirmed the organism as *Phytobacter* species. The patient was managed with blood component transfusions, filgrastim, and empirical piperacillin–tazobactam. Antimicrobial therapy was stopped on day 8, with recovery of blood counts noted by day 7. Subsequently, chemotherapy was resumed with trastuzumab and single-agent taxane at a reduced dose.

**Conclusion:**

Gram-negative infections caused by phytobacteria are likely underreported with the automated VITEK-2 identification system. This first molecularly confirmed case of *Phytobacter diazotrophicus* identified by 16S rDNA sequencing in India underscores the need for heightened clinical awareness, prompt and accurate microbiological identification, and vigilance regarding antimicrobial resistance, especially in the immunocompromised population.

## Background

*Phytobacter diazotrophicus*, though originally recognized as a plant growth-promoting Gram-negative bacterium, belongs to the Enterobacterales species and has recently been identified as an opportunistic pathogen associated with nosocomial infections (1). This species, as well as the associated genus of *Phytobacter,* was originally described by Zhang et al. in 2008, from wild rice in China, when it was noticed that *P. diazotrophicus* helps promote plant growth via nitrogen fixation (2). Its association with human disease was first described in 2018 in a retrospective analysis of preserved bacterial strains, which were traced back to multiple Brazilian sepsis outbreaks in 2010, 2013, and 2015. *P*. *diazotrophicus* has also been identified as the culprit behind several multidrug-resistant nosocomial infections (3). Globally, *P*. *diazotrophicus* has been isolated from cases of neonatal sepsis or as a contaminant in total parenteral nutrition (TPN) and in the elderly with a compromised immune system. Herein, we report a case of febrile neutropenia caused by *P. diazotrophicus* bloodstream infection in an elderly breast cancer patient following chemotherapy-induced neutropenia. Initial misidentification by the VITEK-2 system highlights the diagnostic challenges posed by this organism and underscores the importance of confirmatory molecular methods such as 16S rDNA sequencing. The emergence of such nosocomial pathogens represents a growing threat to antimicrobial resistance and poses a significant risk to immunocompromised populations, particularly patients with malignancies. To the best of our knowledge, this is the first reported case from India.

## Case presentation

A 62-year-old woman, receiving neoadjuvant chemotherapy with trastuzumab (8 mg/kg loading dose followed by 6 mg/kg), carboplatin (AUC 6), and docetaxel (75 mg/m^2^) for HER2-positive carcinoma of the right breast, stage cT4bN2M0, presented to the hospital on day 12 of the third cycle of chemotherapy with fever, headache, and severe fatigue. On examination, she was conscious, febrile, and had tachycardia (pulse 110/min), severe pallor, and retinal hemorrhages on ophthalmoscopy. Her blood pressure was 130/70 mm Hg, and the systemic examination was normal. On admission, her hemoglobin was 3.7 g/dL, total leucocyte count (TLC) was 1.05 × 10^9^/L, absolute neutrophil count (ANC) was 0.294 × 10^9^/L, and platelet count was 5 × 10^9^/L. Thus, she was diagnosed to have chemotherapy-induced febrile neutropenia (common terminology criteria of adverse events (CTCAEs), grade III). Biochemical parameters, including serum lactate, were normal. Chest X-ray was normal. Blood and urine culture samples were sent, and broad-spectrum IV antibiotic therapy was immediately started with piperacillin-tazobactam (administered as an extended infusion over 3 h) and teicoplanin as per institutional protocol. She also received an injection of filgrastim 300 μg daily to aid neutrophil recovery and transfusion support with packed red blood cells and platelets.

**Figure 1:**
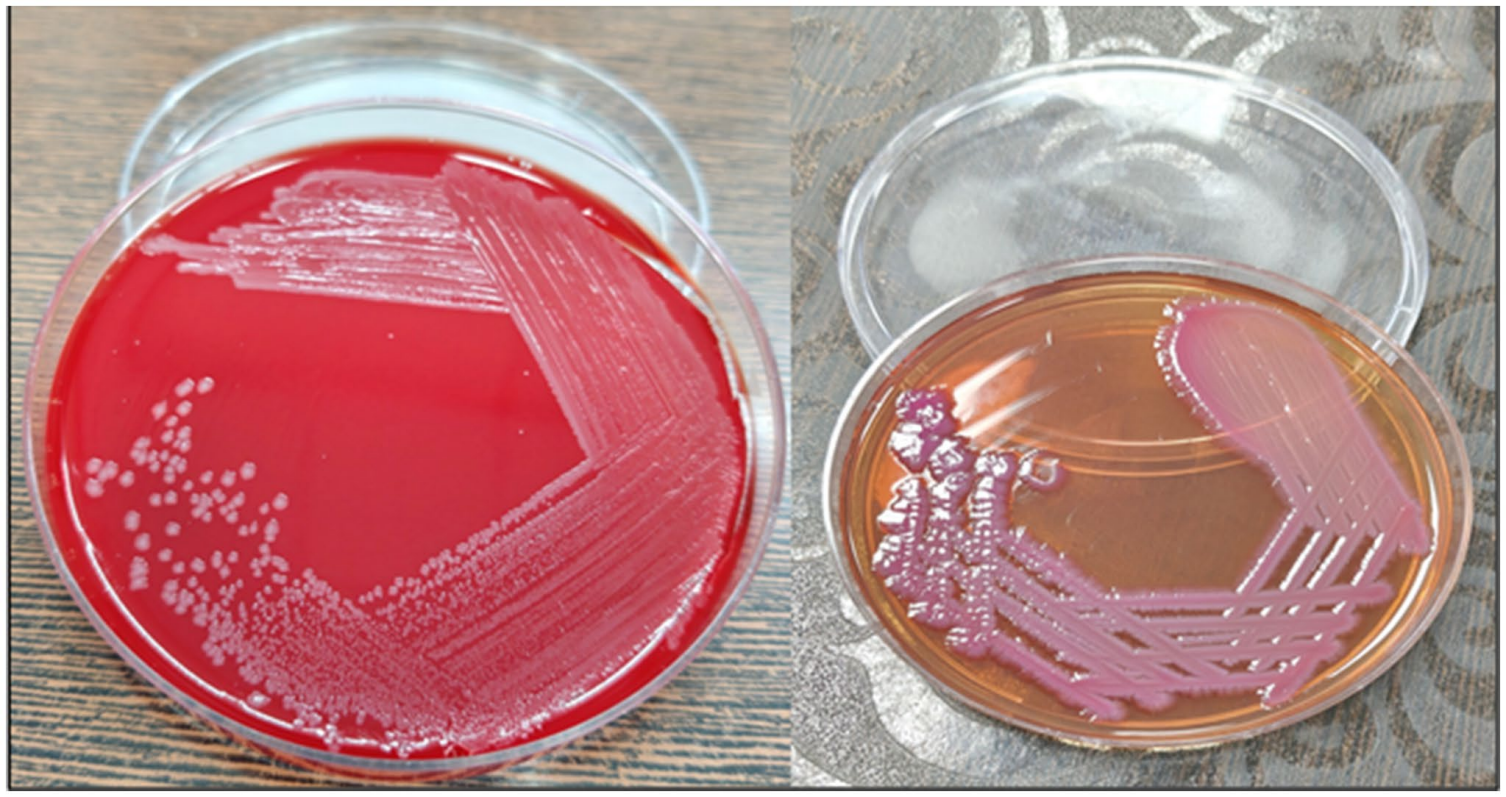
Blood agar and MacConkey agar.

**Figure 2:**
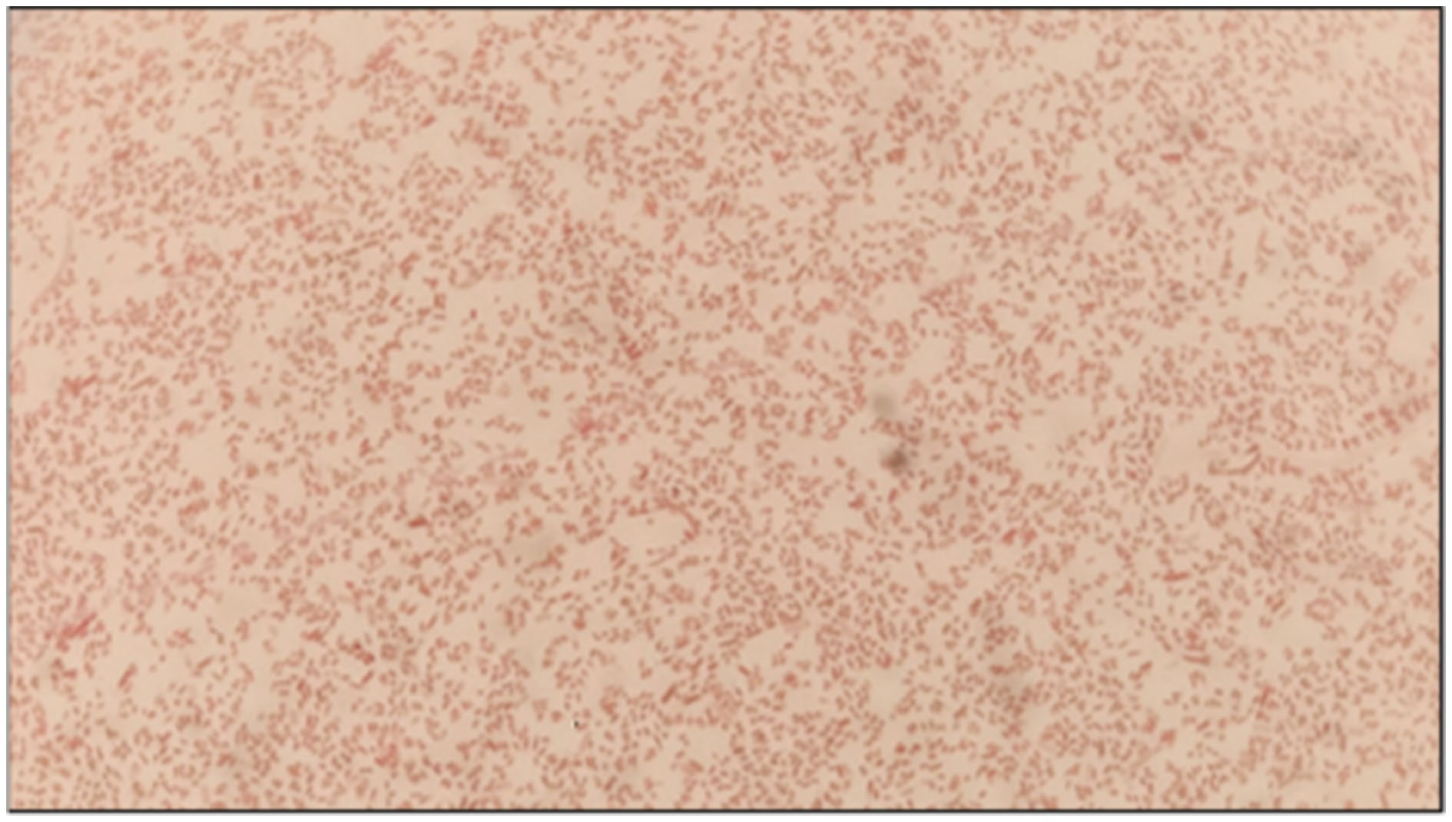
Gram staining of *Phytobacter* sp.

**Figure 3:**
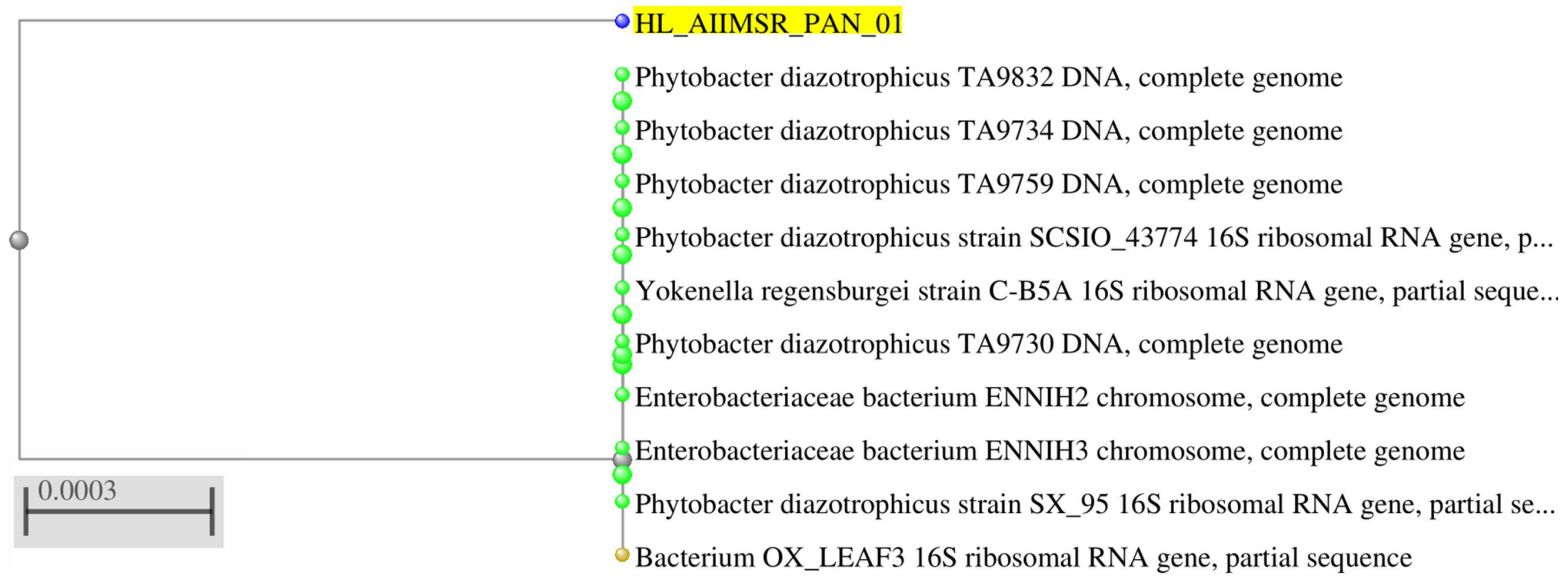
Phylogenetic analysis of *Phytobacter* sp. based on the 16S rDNA gene.

The blood samples were sent in a BacT/ALERT bottle (BioMérieux) for microbiological investigation. It flashed “yes” following a day of incubation. Non-lactose fermenting colonies were cultivated by subculturing the bottle onto blood agar (BA) and Mac-Conkey agar (MAC), as shown in Figure 1. Phenotypic and antimicrobial susceptibility testing (AST) utilizing the automated VITEK-2 (Software:9.04) identification system (BioMérieux, France) initially recognized the isolate as *Pantoea*. For molecular-level identification, 16S rRNA gene sequencing was performed. Genomic DNA was first isolated, followed by PCR amplification using universal primers (forward primer: 5′-CCTACGGGNGGCWGCAG-3′and reverse primer: 5′-GACTACHVGGGTATCTAATCC-3′; Figure 2). The amplified products were sequenced using the Illumina MiSeq platform with a 2 × 300 bp paired-end V3–V4 sequencing kit. Sequence data were analyzed, and strain identification was carried out using the NCBI database. 16S rDNA sequence analysis indicated that the isolate was closely related to Phytobacter sp. (Figure 3). To achieve species-level identification, average nucleotide identity (ANI) analysis was conducted (4), and the resulting ANI values are presented in Table 1. Genome-based ANI analysis showed that the newly isolated clinical strain exhibited ANI values below the species threshold (95–96%) for all Phytobacter species except *Phytobacter diazotrophicus*, indicating a close relationship with *P. diazotrophicus* (5).

**Table 1:** ANI values against the other *Phytobacte*r sp.

Strain name	ANI value
*Phytobacter diazotrophicus* strain DSM 17806	98.88
*Phytobacter diazotrophicus* strain TA9730	98.99
*Phytobacter diazotrophicus* strain TA9832	98.97
*Phytobacter massiliensis* isolate MGYG-HGUT-01426	80.8
*Phytobacter ursingii* strain OUH-01	91.99
*Phytobacter ursingii* strain CAV1151	91.76
*Pantoea dispersa* strain VWJL. P1	70

The minimal inhibitory concentration (MIC) values and their justifications are summarized in Table 2. The results of the antibiotic susceptibility test (AST) were reported in accordance with the 2025 edition (M-100 S35 edition) MIC breakpoint recommendations published by the Clinical and Laboratory Standards Institute.

**Table 2:** AST profile of *Phytobacter* spp.

Name of antibiotics	MIC values	Interpretation
Amikacin	≤1	S
Gentamycin	≤1	S
Imipenem	≤0.25	S
Meropenem	≤0.25	S
Cefepime	≤0.12	S
Cefoperazone/Sulbactam	≤8	S
Piperacillin/tazobactam	≤1	S
Amoxicillin/Clavulanic acid	≤0.2	S
Trimethoprim/sulfamethoxazole	≤20	S
Ciprofloxacin	≤0.06	S
Colistin	≤0.5I	I
Cefuroxime	≤1	S
Ertapenem	≤0.12	S
Ceftriaxone	≤0.25	S

**Figure 4:**
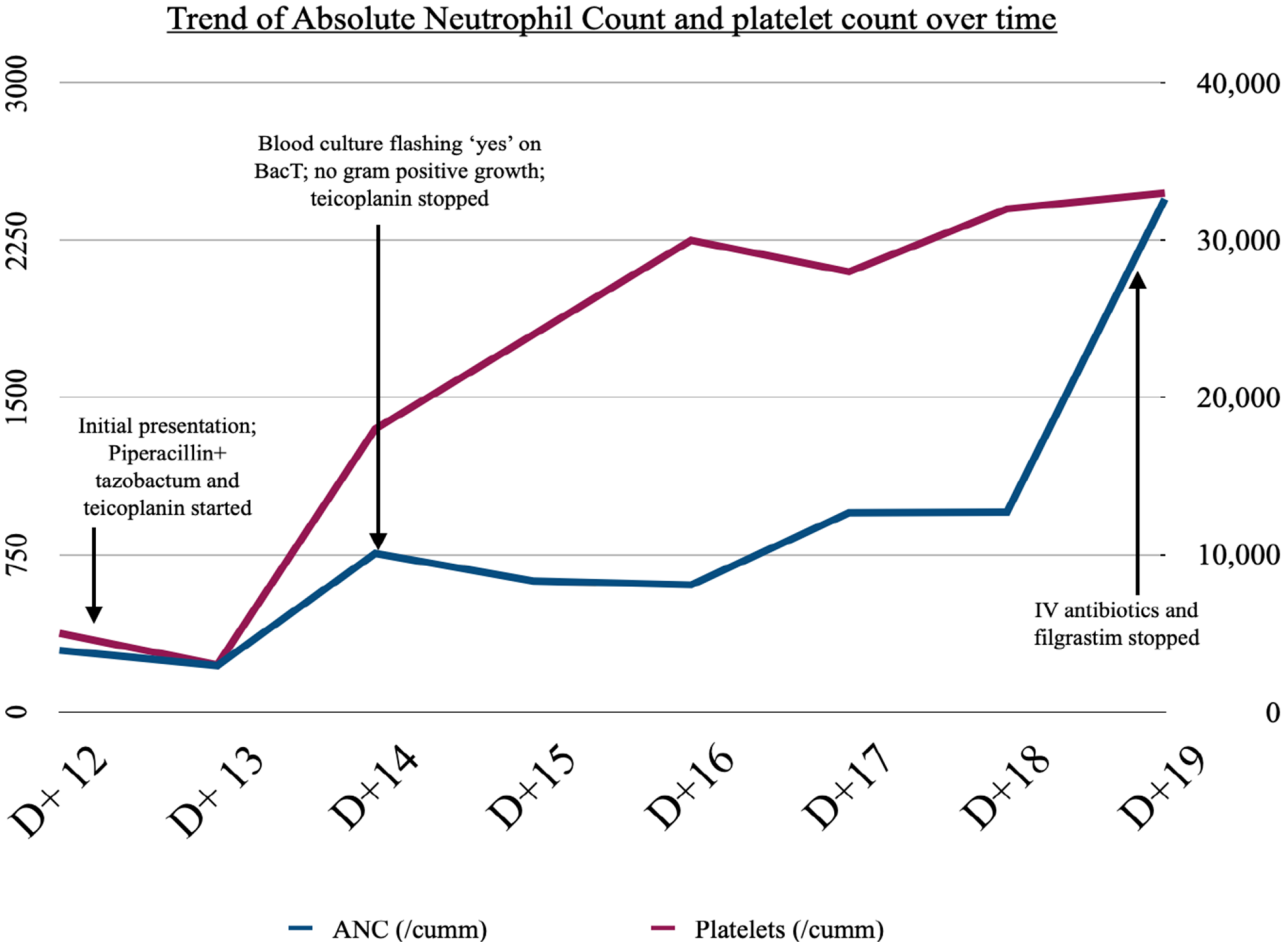
Timeline of clinical events and trend of ANC and platelet count.

In view of the lack of Gram-positive growth and an overall improvement in the general condition of the patient, teicoplanin was stopped on day 3 of hospitalization. Piperacillin-tazobactam was continued along with transfusion support and filgrastim. Fortunately, the isolate in our patient was sensitive to most of the antibiotics, including cefepime, ertapenem (MIC ≤0.12), meropenem, and ceftriaxone (MIC ≤0.25), among the rest. There was a complete recovery from pancytopenia on day 7 of hospitalization. Antibiotics were stopped, and the patient was discharged on day 8. Patient subsequently followed up in our outpatient and continued to receive three additional cycles of neo-adjuvant chemotherapy with trastuzumab and single-agent reduced-dose taxane. She did not experience any grade III/IV toxicities and tolerated the rest of her treatment well. She also underwent a right modified radical mastectomy with a complete pathological response and is currently receiving adjuvant trastuzumab. A timeline depicting the clinical course is given in Figure 4.

## Discussion

The genus *Phytobacter* belongs to the family Enterobacteriaceae and is characterized by soil-borne diazotrophic species with an endophytic lifestyle, found in association with several plant species such as rice (*Oryza rufipogon*), oil palm, sugarcane, or switchgrass. It includes four species*: Phytobacter diazotrophicus*, *Phytobacter ursingii*, *Phytobacter palmae,* and *Phytobacter massiliensis* (6). In recent years, reports of multidrug-resistant *P. diazotrophicus* have been increasing. Most of these cases have been reported from neonatal intensive care units after the use of intravenous fluids or medical devices. Lin et al. isolated this pathogen from a neonatal sepsis patient (7). Another case series from Argentina included two elderly immunocompromised patients and one neonate, in whom the initial phenotypic identification using conventional biochemical tests was compatible with *Pantoea* spp. Species identification using matrix-assisted laser desorption ionization-time of flight mass spectrometry yielded *P. ursingii* in the neonate and *Phytobacter* sp. in the other three patients. On whole-genome sequencing, these were then identified to be *P. diazotrophicus* (8).

The VITEK 2 system identifies bacteria using biochemical reactions such as sugar fermentation and enzyme activity. *Phytobacter* and *Pantoea* share highly similar biochemical profiles, as both are Gram-negative, oxidase-negative, and ferment glucose, resulting in nearly identical metabolic signatures on standard VITEK cards (9). Since *Phytobacter* spp. are strong lactose-fermenting Gram-negative bacilli, they may resemble *E. coli* and *Citrobacter* on EMB Levine agar. Additionally, negative results for lysine decarboxylase, ornithine decarboxylase, and arginine dihydrolase can lead to the misidentification of these strains as *Pantoea* spp. or *Pantoea agglomerans*, as was seen in our patient. This has led to an underestimation of *Phytobacter* spp. in fatal outbreaks (8). Molecular methods (10) are essential for resolving ambiguous bacterial isolates. Sequencing of conserved genes such as 16S rRNA, or preferably gyrB, improves species-level discrimination within the Enterobacteriaceae. Although whole-genome sequencing provides the highest resolution—particularly in outbreak investigations through analyses such as ANI, dDDH, and core-genome phylogeny—its limited availability restricts its routine use. Consequently, accurate identification of Phytobacter spp. remains challenging in clinical microbiology laboratories.

Currently, several case reports and series have confirmed the occurrence of *Phytobacter* spp. As of July 2024, there were 41 draft or complete genomes of *Phytobacter* spp. listed at NCBI, of which no less than 25 were of direct human or clinical origin (6). In addition, several case reports describing multidrug-resistant strains of *Phytobacter* spp. have been published. Worryingly, these strains carried carbapenem resistance genes, bla_NDM − 1_ or bla_KPC_, on plasmids resistant to most *β*-lactam antibiotics (1, 11). Fortunately, our patient did not harbor any such strains and responded to piperacillin-tazobactam, which was given as an extended infusion over 3 h to achieve maximum therapeutic benefits in our neutropenic patient. Though *Phytobacter* spp. are commonly associated with the use of contaminated TPN formulations, our patient had not received any form of TPN and did not have a central venous catheter. The current episode in our patient appears to be directly related to the immunocompromised state due to chemotherapy-induced neutropenia.

For the treating physician, early identification of the causative pathogen and initiation of appropriate antibiotics is of utmost importance. This case report highlights the need to be aware of the occurrence of *Phytobacter* spp., which is not only notorious for causing nosocomial outbreaks but may also lead to delayed diagnosis and treatment due to diagnostic conundrums.

## Conclusion

*Phytobacter* spp. are responsible for outbreaks of Gram-negative infections in the neonatal units and among those with a compromised immune system. However, their true global impact has been underestimated due to misidentification and inherent limitations in the testing procedures. This is the first case report from India with 16S rDNA sequencing-confirmed *Phytobacter diazotrophicus* bloodstream infection in a patient with febrile neutropenia. The emergence of multidrug-resistant strains can pose serious problems for the clinician, and a high index of suspicion is required for timely diagnosis and intervention.

## Data Availability

The original contributions presented in the study are included in the article/supplementary material, further inquiries can be directed to the corresponding authors.
